# Hyperfine Structure Constants for Diatomic Molecules

**DOI:** 10.6028/jres.103.014

**Published:** 1998-04-01

**Authors:** I. Tupitsyn, S. Kotochigova

**Affiliations:** Physics Department, St. Petersburg University, St. Petersburg, Russia, 198904; National Institute of Standards and Technology, Gaithersburg, MD 20899-0001

**Keywords:** AgH^+^ molecule, diatomic molecules, Fermi contact term, hyperfine structure, OH molecule, valence-bond method

## Abstract

The multiconfiguration valence-bond method (VB) is applied to diatomic molecules using the Hartree-Fock (HF) atomic basis set. The hyperfine constant, Fermi contact term, is computed as a function of the interatomic separation for the X^2^∏ ground state of ^17^OH and X^1^∑ ground state of ^107^AgH^+^ molecules. This study leads to a number of conclusions about the influence of correlation and polarization effects on the hyperfine structure of hydrogenic molecules. The calculated values of the Fermi contact term are found to agree within 1 % of the experimental values wherever available.

## 1. Introduction

In this work, the hyperfine Fermi contact term as a function of internuclear distance *R* is computed for the X^2^∏ state of OH and the X^2^∑ state of AgH^+^. The all electron *ab initio* valence-bond method [1] with Hartree-Fock (HF) and Sturm’s [2] basis sets is used to calculate the electron spin density near the nuclei correctly. This leads to an accurate determination of the molecular magnetic dipole coupling constants and in particular the molecular Fermi contact term, *A*_c_. The method uses many-electron atomic wave functions to construct the molecular wave function and yields the correct asymptotic properties for the molecule, one of which is the convergence of the molecular hyperfine interaction parameters to the atomic values for large internuclear separations.

Magnetic hyperfine parameters are very sensitive to the quality of the molecular wave function in general and to the spin polarization of atomic cores in particular. One of these parameters, the Fermi contact term, is proportional to the electron spin density at the position of the nuclei that have nonzero spin. When a molecule has electrons in open shells, it leads to one or more unpaired spins in valence orbitals and gives an unbalanced spin density at the nuclei. Computationally it implies different exchange potentials for electrons with spin up and spin down. The contact term, *A*_c_, is proportional to the difference in the spin densities of electrons with the opposite direction of spin. The accurate determination of this parameter requires both correlation and polarization interactions in the model. The configuration interaction (CI) approach is used to treat these effects. We build the CI on the basis of the nonorthogonal many-electron atomic HF and Sturm’s functions.

Hyperfine splittings have been observed in the optical spectra of diatomic molecules with very high accuracy. Very precise theoretical calculations are required for comparison. One of these calculations was performed by Kristiansen and Veseth [3] for the OH molecule. A many-body perturbation theory was used to compute magnetic hyperfine parameters for the lowest vibrational levels of the ^2^∏ ground state. Their disagreement with experiment does not exceed 2 % near the equilibrium distance *R*_e_ = 1.8342 a.u. (the atomic unit of length, a.u., is the Bohr radius *a*_0_ and is approximately equal to 0.0529177 nm).

In our calculations the Fermi contact term is determined over a wide range of *R* (1.5 a.u. to 8 a.u.). The asymptotic value of *A*_c_ can be compared with our value at the largest internuclear separations.

The only previous study of the molecular potentials of the AgH^+^ molecule [4] was performed using a pseudopotential approach with its inherent integrated treatment of the core orbitals, and therefore does not give theoretical information about the AgH^+^ hyperfine coupling constants. Our *A*_c_(*R*) values were obtained for *R* between 2 a.u. and 18 a.u. and converge for large *R* to the atomic values.

## 2. Theoretical and Computational Details

The total electronic wave function *Ψ*_AB_ of the molecule is introduced as a linear combination of Slater’s determinants *det*_α_, corresponding to various configurations α of atoms A and B. That is

$$\psi_{\text{AB}}=\sum_{\alpha }C_{\alpha }{det}_{\alpha }$$ (1)

where the *C*_α_ are obtained by solving a generalized eigenvalue matrix problem, described by the equation

$${\hat{H}}_{\text{AB}}C={\hat{S}}_{\text{AB}}C,$$ (2)

where ${\hat{H}}_{\text{AB}}$ is the Hamiltonian of the molecule AB. The right-hand-side of Eq. (2) includes the nonorthogonality matrix ${\hat{S}}_{\text{AB}}$, which describes an overlap between determinants <*det*_α_|*det*_β_> and it is given as

$${\left({\hat{S}}_{\text{AB}}\right)}_{\alpha \beta }=<{det}_{\alpha }|{det}_{\beta }>={\left(D_{\alpha \alpha }D_{\beta \beta }\right)}^{-1/2}D_{\alpha \beta },$$ (3)

*D*_αβ_ = *det*|< α_1_|β_1_ > … < α*_i_*|β*_j_* > … < α*_N_*|β*_N_* > |, and *N* is the total number of electrons in the molecule. For the particular one-electron orbitals α*_i_* and β*_j_*, the overlap matrix elements ${\hat{S}}_{i,j}^{\alpha \beta }$ are used to describe the one-electron density matrix $\rho_{1}^{\alpha ,\beta }(x,x^{\prime })$ of the molecule as

$$\begin{array}{c} \rho_{1}^{\alpha ,\beta }(x,x^{\prime })={\left(D_{\alpha \alpha }D_{\beta \beta }\right)}^{-1/2}D_{\alpha \beta }\\ \sum_{i,j}^{N}{\left(S^{-1}\right)}_{i,j}^{\alpha ,\beta }\cdot \phi_{i}(x)\cdot \phi_{j}^{*}(x^{\prime }), \end{array}$$ (4)

where the *ϕ*(*x*) are the one-electron wave functions and *x* denotes both coordinates and spin of the electron.

Moreover, the two-electron density matrix is

$$\begin{array}{c} \rho_{2}^{\alpha ,\beta }(x_{1},x_{2}|x_{1}^{\prime },x_{2}^{\prime })={\left(D_{\alpha \alpha }D_{\beta \beta }\right)}^{-1/2}\cdot D_{\alpha \beta }\\ \sum_{i,j}^{N}\sum_{k,l}^{N}D_{i,j,k,l}^{\alpha \beta }\cdot \phi_{i}(x_{1})\phi_{j}^{*}(x_{1}^{\prime })\phi_{k}(x_{2})\phi_{1}^{*}(x_{2}^{\prime }), \end{array}$$ (5)

where

$$D_{i,j,k,l}^{\alpha \beta }=D_{\alpha \beta }\cdot [{\left(S^{-1}\right)}_{i,j}^{\alpha ,\beta }\cdot {\left(S^{-1}\right)}_{k,l}^{\alpha ,\beta }{\left(S^{-1}\right)}_{i,1}^{\alpha ,\beta }\cdot {\left(S^{-1}\right)}_{k,j}^{\alpha ,\beta }]$$ (6)

Finally, the Fermi contact term can be expressed in terms of the total spin densities *ρ*^α,β^(↑) and *ρ*^α,β^(↓) at each nuclear site for the electrons with spin pointed up (↑) and down (↓). The CI form of *A*_c_ is introduced as

$$A_{c}=2\;\mu_{0}g_{I}\mu_{N}\sum_{\alpha ,\beta }\frac{8\pi }{3}c_{\alpha }c_{\beta }[\rho^{\alpha ,\beta }(↑)-\rho^{\alpha ,\beta }(↓)]$$ (7)

where *g*_I_ denotes the nuclear *g*-factor, *μ*_N_ is the nuclear magneton, and *μ*_0_ is the Bohr magneton.

**Fig. 1:**
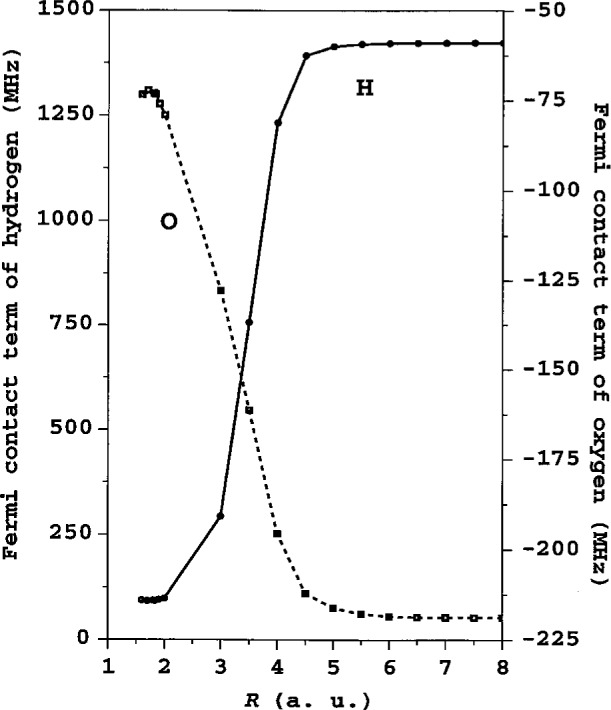
Fermi contact term *A*_c_ of the ^17^OH molecule as a function of the internuclear separation (1 a.u. = 0.0529177 nm).

In our model the CI expansion for OH includes configurations obtained from the occupied 1*s*^2^, 2*s*^2^, and 2*p*^4^ shells of oxygen and the 1*s* shell of hydrogen, as well as configurations created by excitations of these electrons into the 3*s*, 3*p*, 3*d*, and 4*s* states for O and the 2*s*, 2*p*, 3*s*, 3*p*, and 3*d* for H. The total number of configurations was equal to 238. To describe the virtual excited states we use localized Sturm’s functions. The result of our computation of the *A*_c_ (*R*) for the oxygen and hydrogen atom in the ^17^OH molecule is shown in Fig. 1. The agreement of the calculated values over the wide range of internuclear separations *R* (1.5 a.u. to 8 a.u.) is within less than 1 % of the precise experimental values, which are for the equilibrium distance *R*_e_ = 1.8342 a.u., *A*_c_ = −73.1258 MHz [5], and for the asymptotic values *A*(O) = −219.6 MHz and *A*(H) = 1420.40575 MHz [6, 7].

**Fig. 2:**
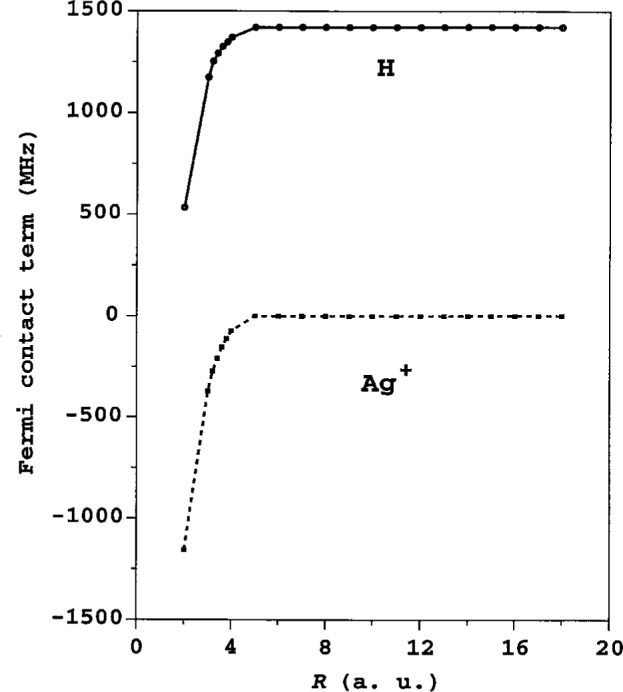
Fermi contact term *A*_c_ of the ^107^AgH^+^ molecule as a function of the internuclear separation (1 a.u. = 0.0529177 nm).

The CI treatment of the AgH ^+^ molecule was based on the occupied 4*p*^6^4*d*^10^ shells for the Ag^+^ ion and the 1*s* shell for the H atom as well as the 5*s*, 5*p*, 5*d*, 6*s* and 2*s*, 2*p*, 3*s*, 3*p*, 3*d* virtual Sturm’s orbitals for Ag^+^ and H, respectively. Computed values of the Fermi contact term of 107AgH^+^ molecule are presented in Fig. 2. The asymptotic values of *A*_c_ for H and Ag^+^ fit with about 1 % disagreement to the well known experimental value of the hyperfine coupling constant for the H atom, and to the expected zero value for the closed shell ion Ag^+^.
